# Metabolic Profiling and Transcriptome Analysis Reveal the Key Role of Flavonoids in Internode Coloration of *Phyllostachys violascens* cv. Viridisulcata

**DOI:** 10.3389/fpls.2021.788895

**Published:** 2022-01-28

**Authors:** Han-tian Wei, Dan Hou, Muhammad Furqan Ashraf, Hai-Wen Lu, Juan Zhuo, Jia-long Pei, Qi-xia Qian

**Affiliations:** ^1^State Key Laboratory of Subtropical Silviculture, Zhejiang A&F University, Lin’An, China; ^2^College of Landscape Architecture, Zhejiang A&F University, Lin’An, China

**Keywords:** bamboo, internode coloration, metabolic, transcriptome, flavonoids

## Abstract

Bamboo, being an ornamental plant, has myriad aesthetic and economic significance. Particularly, *Phyllostachys violascens* cv. Viridisulcata contains an internode color phenotype in variation in green and yellow color between the sulcus and culm, respectively. This color variation is unique, but the underlying regulatory mechanism is still unknown. In this study, we used metabolomic and transcriptomic strategies to reveal the underlying mechanism of variation in internode color. A total of 81 metabolites were identified, and among those, prunin as a flavanone and rhoifolin as a flavone were discovered at a high level in the culm. We also found 424 differentially expressed genes and investigated three genes (*PvGL*, *PvUF7GT*, and *PvC12RT1*) that might be involved in prunin or rhoifolin biosynthesis. Their validation by qRT-PCR confirmed high transcript levels in the culm. The results revealed that *PvGL*, *PvUF7GT*, and *PvC12RT1* might promote the accumulation of prunin and rhoifolin which were responsible for the variation in internode color of *P. violascens*. Our study also provides a glimpse into phenotypic coloration and is also a valuable resource for future studies.

## Introduction

Bamboo (Bambusoideae) belongs to the Poaceae and is a woody grass broadly cultivated in the temperate, subtropical, and tropical regions between 46° N and 47° S. China is the central place of cultivation and is also known as the “Kingdom of Bamboo,” with more than 34 genera and 534 species (McDowell et al., 2015; Liu et al., 2018; Cai et al., 2019). As a non-timber forest resource, bamboo can be processed into many products, such as furniture, paper, and various crafts (Sharma et al., 2015). Around 2.5 billion people are directly producing and consuming bamboo, and its international trade reached US**$** 68.8 billion (Ye et al., 2020). Bamboo species are designated as “green gold,” with myriad cultural, aesthetic, economic, and technology development significance, and have a special place among traditional ornamental plants in garden landscapes in Asia (Zheng et al., 2021).

Naturally, the colorful internode shape is special and stable among few bamboo species. For instance, *Phyllostachys violascens* cv. Viridisulcata exhibits variation in internode color and is recognized as an ornamental plant in China (Lin et al., 2008, 2011). Bamboo internode color variation is very common in nature, as reported in *P. vivax* cv. Aureocaulis (Xia et al., 2015) and *P. edulis* cv. Tao Kiang (Jin and Yuan, 2011). Interestingly, there exists a stable and special type of internode color variation in nature, the green sulcus with yellow culm, respectively. This color pattern occurs in many bamboo types, such as *P. aureosulcata* cv. Spectabilis, *P. sulphurea* cv. Robert Young, and *P. violascens* cv. Viridisulcata (Lin et al., 2011; Recht and Wetterwald, 2015). The coloration attribute of plants is associated with pigment biosynthesis, catabolism, and finally accumulation in determining the plant appearance (Allan et al., 2008). The main pigments in plants are flavonoids, chlorophylls, carotenoids, and betalains that can help the plants to develop their characteristic color (Tanaka et al., 2008; Cazzonelli, 2011; Gandia-Herrero and Garcia-Carmona, 2013; Zepka et al., 2019). However, the underlying regulatory mechanism of bamboo internode pigmentation remains poorly unknown.

Flavonoids are among the key secondary metabolites present in different plants; they create color variations with different shades such as pale-yellow to blue and are synthesized within plant parts (e.g., leaves, bark, stems, flowers, and seeds) and also participate in the plant defense against stressors (Koes et al., 2005; Carrier et al., 2013; Panche et al., 2016). Flavonoids can be subdivided into different subgroups, and there are flavones, flavonols, flavanones, flavanonols, flavanols or catechins, anthocyanins, and chalcones (Cassidy and Minihane, 2017; Shi et al., 2021). During early flavonoid biosynthesis, phenylalanine precursor is first delivered to cinnamic acid by the action of phenylalanine-ammonia lyases (PAL) and then to chalcone synthase (CHS), catalyzing the reactions using the three and one units of malonyl-CoA and CoA-ester, respectively, to produce naringenin chalcone (Tanaka et al., 2008). Flavanone (Ferrer et al., 2008; Vogt, 2010) is generated by the isomerization of chalcone *via* chalcone flavanone isomerase (CHI). As the subgroups of flavonoids, flavanone and flavone have a common naringenin chalcone intermediate to produce prunin and rhoifolin using naringenin and apigenin, respectively (Refaat et al., 2015; Jung et al., 2017). Apigenin is converted to comosin by flavone-7-*O*-beta-glucosyltransferase (UF7GT). Rhoifolin synthesis uses the formal reaction product (i.e., comosin) by flavanone-7-*O*-glucoside 2″-*O*-beta-l-rhamnosyltransferase (C12RT1) (Kumar et al., 2013; Lou et al., 2014). Prunin is also generated from naringenin by flavanone-7-*O*-beta-glucosyltransferase (GL) before naringin, which is considered the final metabolite (Tao et al., 2017).

In *Arabidopsis*, R2R3-MYB proteins MYB11, MYB12, and MYB111 regulated early flavonoid biosynthetic steps by activating the early flavonoid biosynthetic genes CHS, CHI, F3H, and FLS1 (Mehrtens et al., 2005; Stracke et al., 2007; Zhou et al., 2019). Several late flavonoid biosynthetic genes, such as *DFR*, *LODX*, *ANR*, and *TT2*, are activated by the MYB-bHLH-WD40 (MBW) ternary transcriptional complex comprising three classes of regulatory proteins, including R2R3-MYBs, bHLHs, and WD40s (Nesi et al., 2002; Appelhagen et al., 2011). In addition, TCP3 could interact with R2R3-MYB proteins and promote flavonoid biosynthesis (Li and Zachgo, 2013).

Currently, transcriptome and metabolome technologies greatly help in unmasking the hidden facts behind the phenotypes in plants. Many researchers have used these technologies to understand the color mutations/variations in different types of plants and/or parts (Tanaka et al., 2008; Wang et al., 2010; Glover and Martin, 2012; Karlova et al., 2014). For instance, the possible molecular mechanisms of litchi fruit surface coloration were determined using the transcriptome approach to obtain litchi pericarp-related data (Tanaka et al., 2008). Similarly, data analysis of the metabolome and transcriptome of peel color (green and purple) changes in fig fruit revealed the novel flavonoids and the other genetic factors that regulate peel color variation (Wang et al., 2017). Studies on color changes have found, in addition to the abovementioned flavonoids, that genes encoding enzymes participate in modulating the biosynthesis of secondary metabolites and color variations. However, the pattern-related coordination networks of bamboo internode color change and paths that metabolites and genes/TFs adopt for a transcription regulation mechanism remain to be elucidated.

In this study, we used metabolome as well as transcriptome comparisons of the green sulcus and yellow culm of *P. violascens* to reveal the internode color pattern in bamboo. The results suggested that *PvGL*, *PvUF7GT*, and *PvC12RT1* may influence bamboo internode coloration by regulating the accumulation of prunin and rhoifolin. Our studies also support a valuable resource about metabolome and transcriptome analysis for exploiting the possible regulatory mechanism about bamboo internode coloration.

## Materials and Methods

### Plant Materials

*Phyllostachys violascens* was cultivated and maintained in the Bamboo Garden of Zhejiang Agriculture and Forestry University (ZAFU), Zhejiang Province, China (30°15′N, 119°43′E). The cultivated place belongs to the mid subtropical monsoon climate zone with four distinct seasons. The annual rainfall is 1,628.6 mm, and the annual average temperature is 16.4°C. We set up three biological replicates for sampling that had uniform sizes at the height of 3 m. Sampling was carried out from the middle of a bamboo plant (with maximum internode length for the representative samples) on April 24, 2019. Quickly, all samples were sterilized with 75% ethanol and stored in liquid nitrogen.

### Measurement of Total Flavonoid Contents

The spectrophotometric method was used to determine total flavonoid contents (Shi et al., 2012; He et al., 2018). In brief, the rutin solution was prepared using methanol and anhydrous rutin after ultrasonic mixing, which was used as a reference solution. The volume of the 70% methanol solution was 10 ml for extracting the flavonoids using the ground sample, and then samples were placed into a hot water bath at 70^°^C for 20 min. After extracting, the sample solution was centrifuged at 4,000 × *g* for 10 min, and the total 10 ml volume was adjusted using 70% methanol solution. After volume calibration, we used the 1 ml sample solution and added 0.5 ml of 5% NaNO_2_ and 0.5 ml of 10% AlCl_3_ solutions one by one, set for 6 min, then added 4 ml of 4% sodium hydroxide solution, and kept the whole solution for 15 min after shaking. For control or blank, we used the respective reagent solution in the flask. Then, each solution was used to calculate the values with the UV-visible spectrophotometer and led to the standard curve computation. The flavonoid content was calculated using the following linear equation based on the calibration curve: *A* = 2.091 *C*-0.09 (*R*^2^ = 0.999), where *A* is the absorbance and *C* is the flavonoid content in mg*ml^–1^.

### Measurement of Chlorophyll and Carotenoid Contents

Chlorophyll and carotenoid contents were measured using the spectrophotometric method (Lichtenthaler and Buschmann, 2001). The ground samples were extracted in 95% ethanol at room temperature after centrifugation. The absorption of pigments was measured at 665, 645, and 470 nm using a UV-visible spectrophotometer.

### Transcriptomics

For total RNA extraction, we followed the protocol (Hou et al., 2020) as described in the RNAiso Plus Kit (TaKaRa, Beijing, China). The quality and quantity of total RNAs were ensured using the NanoPhotometer spectrophotometer (IMPLEN, California, United States) and electrophoresis (1% agarose gel), respectively. Then, libraries were synthesized using the NEBNext Ultra™ RNA Library Prep Kit for Illumina (NEB, Massachusetts, United States). These constructed libraries were sequenced using the Illumina HiSeq forum to obtain paired-end reads (150 bp). After quality control (QC), transcriptome data were further handled and assembled by following the procedure described by Grabherr et al. (2011) using Trinity version 2.8.4. Assembled results by Trinity were processed using Corset as described by Davidson and Oshlack (2014). To confirm the completeness of the transcriptome assemblies, we used the Benchmarking Universal Single-Copy Orthologs (BUSCO version 5.2.2) program (Waterhouse et al., 2018) by finding the conserved orthologous genes among transcriptome assemblies (Supplementary Figure 4A). Principal component analysis (PCA) involved using two datasets (one from culm and another from sulcus) after using the normalized-read-count values (Supplementary Figure 4B). Then, we annotated the unigenes using the BLASTX function with the E ≤ 10^–5^ threshold value against diversified databases {i.e., NCBI with non-redundant [protein (Nr) and nucleotide (Nt) sequences]}, Kyoto Encyclopedia of Genes and Genomes (KEGG), Swiss-Prot/UniProt, Eukaryotic Orthologous Group (KOG), as well as Gene Ontology (GO) databases (Supplementary Figure 7). Furthermore, significantly differential expression was determined using sequenced reads with the DESeq2 R package (Love et al., 2014) and padj < 0.05 with | log2FoldChange| > 1, which was set as the threshold during data analysis (Supplementary Figure 5 and Supplementary Table 5).

### Metabolomics

All samples were ground into powder using liquid nitrogen. Homogenized samples were treated with *n*-hexane:acetone:ethanol (2:1:1, v/v/v) mixture followed by 30 s vortexing to get a well-mixed extract. This extract was ultrasound-assisted at 25°C for 20 min and then centrifuged at maximum speed (approximately ≥ 14,000 rpm) for 5 min. We repeated this abovementioned step for the supernatant collection that was dried by evaporating liquid under a stream of nitrogen gas. Afterward, 75% methanol was used to reconstitute the residues. Then, the supernatant was obtained by centrifuging the solution to investigate the liquid chromatography (LC) using a tandem-mass-spectrometry (LC-MS/MS) analyzer. LC-MS/MS analyses involved using the Vanquish UHPLC system (Thermo Fisher, Shanghai, China) coupled with an Orbitrap Q Exactive series mass spectrometer (Thermo Fisher, Shanghai, China). Samples were injected onto a Hyperil Gold column (100 mm × 2.1 mm, 1.9 μm) using a 16-min linear gradient at a flow rate of 0.2 ml/min. The eluents for the positive polarity mode were eluent A (0.1% FA in water) and eluent B (methanol). The eluents for the negative polarity mode were eluent A (5 mM ammonium acetate, pH 9.0) and eluent B (methanol). The solvent gradient was set as follows: 2% B, 1.5 min; 2–100% B, 12.0 min; 100% B, 14.0 min; 100–2% B, 14.1 min; 2% B, 16 min. The Q Exactive mass spectrometer was operated in a positive/negative polarity mode with a spray voltage of 3.2 kV, capillary temperature of 320°C, sheath gas flow rate of 35 arb, and aux gas flow rate of 10 arb. Compound Discoverer 3.0 (CD 3.0, Thermo Fisher) was used to process the raw data generated by UHPLC-MS/MS. The main parameters were set as follows: retention time tolerance, 0.2 min; actual mass tolerance, 5 ppm; signal intensity tolerance, 30%; signal/noise ratio, 3; and minimum intensity, 1,00,000. After normalization of peak intensities, we determined the peak alignment by mzCloud^1^ and ChemSpider^2^ database to obtain the accurate qualitative and relative quantitative results. The Pearson correlation between differential metabolites was analyzed by cor () in R language (Supplementary Figure 2) and PCA was also involved between two datasets (Supplementary Figure 3). Metabolites were annotated using different databases (Supplementary Figure 6), such as the KEGG,^3^ HMDB,^4^ and Lipidmaps databases.^5^ The metabolites with VIP > 1 and *p*-value < 0.05 and fold change ≥ 2 or FC ≤ 0.5 were considered to be differential metabolites. About metabolic pathway enrichment, metabolic pathways were considered as enrichment only when the *p*-value of metabolic pathway < 0.05, and metabolic pathways were considered as statistically significant enrichment (Supplementary Table 8 and Supplementary Figure 8).

### Quantitative Real-Time PCR

The expression profiles of the key genes related to the biosynthesis of flavonoids were validated using qRT-PCR; details of the primers are in Supplementary Figure 9 and Supplementary Table 7. Data analysis involved the 2^–ΔΔCT^ method as described by Schmittgen and Livak (2008), and the reference gene was actin expression (Fan et al., 2013; Hou et al., 2021).

### Data Availability

The metabolomic data are available in the MetaboLights repository under the accession number MTBLS3970.^6^ The sequencing raw data and files related to the gene abundance analysis in this study were deposited in NCBI, Gene Expression Omnibus (GEO), and accessible with the GEO Series accession number GSE157799.^7^

## Results

### Phenotype Examination and Analysis

**FIGURE 1:**
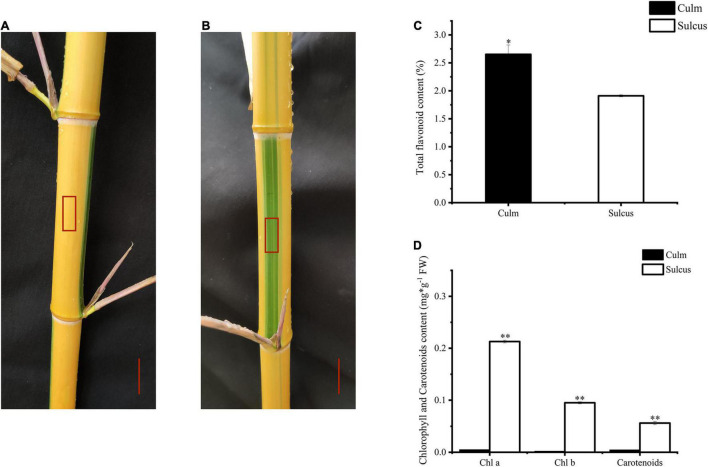
Color phenotype variations in *Phyllostachys violascens* cv. Viridisulcata. Culm **(A)** and sulcus **(B)** in internodes of *P. violascens*, highlighted as a red rectangle. The red scale was 2 cm. Total flavonoid content **(C)** and chlorophyll and carotenoid contents **(D)** in sulcus and culm of *P. violasce*ns. The significant differences were determined by one-way ANOVA with Duncan post-hoc test (*P < 0.05, **P < 0.01).

Globally, bamboo species have been used as ornamental plants. To investigate the changing color pattern of bamboo internodes, we focused on *P. violascens*, which is extensively planted in gardens, to examine the alternating color phenotype differences between culm and sulcus, which are green and yellow, respectively (Figures 1A,B). This unique phenomenon in bamboo implies that pigment variations are affected by the accumulation of various flavonoids. Hence, we found more contents of total flavonoids in the culm as compared to the sulcus (Figure 1C). In contrast, carotenoid and chlorophyll contents were lower in sulcus than culm, which was almost negligible (Figure 1D). From these results, we hypothesized that the accumulation of flavonoids, chlorophyll, and carotenoids might contribute to the internode color changes.

### Metabolome Analysis

Color variations in the culm and sulcus of bamboo internodes imply that metabolic variations are involved in developing phenotypic changes. To test this probability, we used metabolome analysis, after ensuring the quality feature of the non-targeted metabolomics such as the PCA outcomes and strong correlation between the QC samples (Supplementary Figures 2, 3). Our data analysis revealed 846 and 566 metabolites in both tissues under positive and negative ionization modes, respectively. Also, 81 metabolites showed significant (*p* < 0.05) changes in both tissues under the two modes (Supplementary Table 1). Later, annotated data confirmed that most of the metabolites were related to the metabolism of plant secondary metabolites. In this study, we also listed the information of the top twenty differential metabolites that detected higher in culm of *P. violascens* (Supplementary Table 2). In total, 20 and 25 metabolites from positive and negative ionization modes, respectively, were annotated to the global-and-overview maps and were related to the synthesis of secondary metabolites, except for metabolites that also linked to the lipid biosynthesis (Supplementary Figures 6A,B).

### Comparison of Enriched Biological Processes at the Metabolome and Transcriptome Levels

**FIGURE 2:**
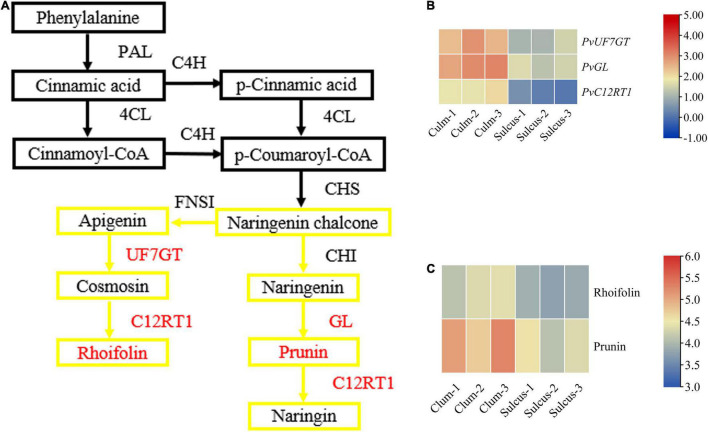
The key genes and metabolites related to the flavonoid pathway. A branch of the flavonoid biosynthesis pathway **(A)** with relative expression of key genes **(B)** and contents of metabolites **(C)**. The red color indicates the differentially expressed genes, and the yellow box indicates the involved flavonoids. Metabolic pathway adapted from Ishihara et al. (2015) and amendments involved displaying the newly identified genes and metabolites under this study and the Kyoto Encyclopedia of Genes and Genomes (KEGG) portal used for analysis.

To estimate the flavonoid biosynthesis pathways that can participate in defining the phenotype of color variation, we focused on the metabolome and transcriptome levels to determine the types of biosynthesis pathways. In this study, short-read sequences were processed using the Trinity program (Supplementary Table 3), and 44,787 unigenes were annotated using the BLASTX against diversified databases (Supplementary Table 4). The expression levels of total unigenes were shown in the volcano plot (Supplementary Figure 5). We functionally characterized the assembled transcripts using the GO and KOG database and provided a description for molecular and biological functions as well as cellular components related to gene products (Supplementary Figures 7A,B). We revealed two distinctive flavonoid responsible pathways, namely, flavonols and flavone, as well as flavonoid biosynthesis, using KEGG abundance analysis at the metabolite level, which displayed a clear significant level among the two tissues (Supplementary Figure 8). In the flavonoid synthesis pathway, we detected faint contents of naringenin, prunin, and rhoifolin in the sulcus. In contrast, the contents of prunin and rhoifolin were visible in culm (Figure 2C). Notably, prunin and rhoifolin belong to the flavonoids that are the major class of the plant secondary metabolites. Our results revealed a clear difference between prunin and rhoifolin contents among both tissues, which might play a critical role in the color phenotype alterations in the internodes of bamboo.

### Flavonoid Biosynthesis May Contribute to Internode Color Variation in Bamboo

The flavonoid pathway is the main secondary metabolism in plants, and the responsible genes encoding the vital enzymes are the central focus in exploiting this novel pathway. We verified that various types of flavonoids accumulate between both tissues of bamboo and help describe the coloration phenotype changes in internodes. Also, we showed the expression of key genes and metabolites involved in the flavonoid synthesis pathway (Figure 2 and Supplementary Table 6), which might participate during internode coloration. The contents of prunin (Com_1145_neg) and rhoifolin (Com_1259_neg) metabolites were high in the culm: 5.37− and 2.86-fold change, respectively (Supplementary Table 1). These results suggested that both metabolites may cause these color variations, being products of the flavonoid pathway. However, we identified only three unigenes involved in prunin and rhoifolin biosynthesis, and their expression was more prominent in culm (Supplementary Table 5). These genes were *PvGL* (Cluster-4806.15107), *PvUF7GT* (Cluster-4806.39584), and *PvC12RT1* (Cluster-4806.1905), and their fold change was 1.47, 1.32, and 5.43, respectively. Additionally, we quantified the expression of *PvGL*, *PvUF7GT*, and *PvC12RT1* using qRT-PCR and analyzed results that were consistent with transcript levels in the culm (Supplementary Figure 9). The expression of *PvPAL1-3* (Cluster-4806.23860, Cluster-4806.23845, and Cluster-4806.23685), *PvCHI1-2* (Cluster-4806.32441 and Cluster-4806.22360), and *PvCHS* (Cluster-4806.23668) were also high but did not show changed expression between sulcus and culm. Overall, our results suggest that the *PvGL*, *PvUF7GT*, and *PvC12RT1* might participate in the biosynthesis pathway of flavonoids, which possibly leads to the accumulation of plant secondary metabolites and the visible internode color variation in bamboo.

### Coexpression Network Between the Metabolites and Genes

**FIGURE 3:**
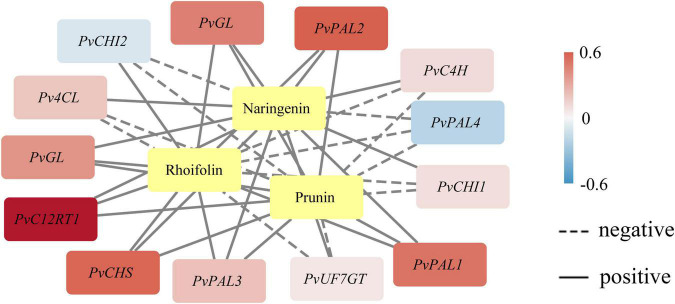
Coexpression analysis of genes related to flavonoid biosynthesis. The yellow color represents metabolites. Pearson correlation coefficients are shown by color range. Correlations between genes and metabolites are designated with black edges.

Phenotype analysis of plants in which certain metabolites and transcripts positively or negatively establish the coexpression network is valuable for illuminating the key characteristic function or trait (Hou et al., 2022). We demonstrated an integrated network using the metabolites and the expression profile of the novel genes under study by Pearson product-moment correlation analysis (Figure 3). The coexpression networks indicated that *PvGL*, *PvPAL1*, *PvPAL2*, *PvC12RT1*, and *PvCHS* expression was highly correlated with naringenin, prunin, and rhoifolin contents. The *PvGL* and *PvC12RT1* genes may be the keys to the variation in bamboo internode color. The expression of the five genes was positively associated with the contents of the three detected metabolites, so these genes might promote flavonoid biosynthesis.

## Discussion

Recently, color variations in plants are in great demand and remain the pivotal concern to increase the aesthetic value of a place. Being an ornamental plant, *P. violascens* cv. Viridisulcata contains a colored internode culm. To disclose the regulation mechanism of colored internode in bamboo, it may facilitate genome editing to create more diverse ornamental bamboos. We used the transcriptomic and metabolomic approaches (Supplementary Figure 1) to reveal the key metabolites and genes that participate in coloration in bamboo.

This study reveals more accumulation of total flavonoids in bamboo culm, especially the contents of prunin and rhoifolin, which were expressed significantly during flavonoid biosynthesis in *P. violascens*. Prunin and rhoifolin belong to the flavanone and flavone color classes of flavonoids, respectively. Flavonoids are well-known as pigments, which accumulate in various parts of plants and display colored variations (Iwashina et al., 2008; Tadmor et al., 2010; Feder et al., 2015). Similarly, many studies demonstrated that the flavanone and flavone accumulation in plants may be the cause to introduce yellow color among various parts (Yoshida et al., 2004; Ono et al., 2006; Reis et al., 2018). For example, lemon-yellow sorghum and *Dianthus knappii* (known a source for yellow pigmentation) presented more contents of the same classes of flavonoids (Dykes et al., 2011; Zhang et al., 2021), and the colored peel in *Cucumis melo* also uncovered 10 kinds of significantly different flavanones, one of them was the prunin in yellow melon (Gatt et al., 1998). Therefore, we focused on the flavonoid pathway and speculated that the accumulation of prunin and rhoifolin might be applicable to the yellow culm of *P. violascens*.

Internode coloration attribute is witnessed in the *P. violascens*, and we determined the key genes, which can influence flavonoid biosynthesis. Notably, the flavonoid pathway is well recognized as first UF7GT converts apigenin to comosin, then C12RT1 converts comosin to rhoifolin subsequently, and prunin is catalyzed by GL from naringenin. Many reports also revealed that *GL*, *UF7GT*, and *C12RT1* may play an important role in regulating the synthesis of flavonoid biosynthesis in *Camellia sinensis* (Wang et al., 2012; Zhao et al., 2017), *Arabidopsis thaliana* (Routaboul et al., 2006), and *Lonicera macranthoides* (Chen et al., 2018). Herein, the transcriptome analysis revealed that the encoding enzymes of *PvGL, PvUF7GT*, and *PvC12RT1* genes were responsible for the biosynthesis of the flavonoid pathway in *P. violascens* were affected significantly by altering the expression profile in culm as compared to sulcus (Figure 2).

**FIGURE 4:**
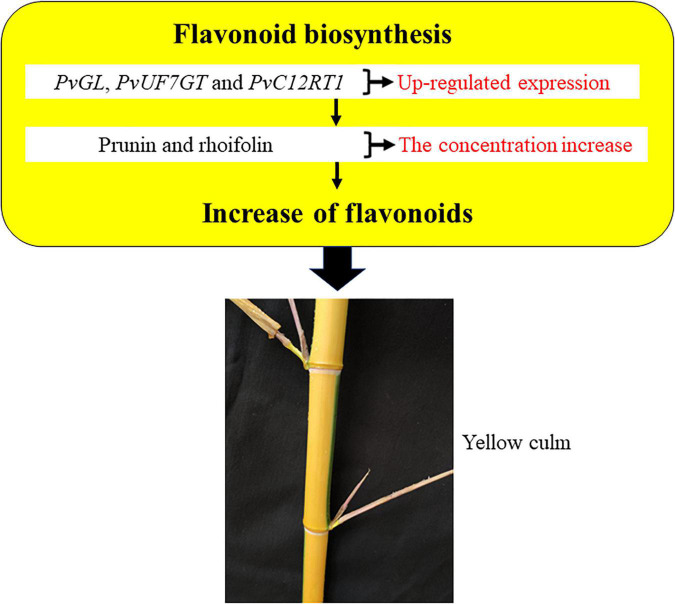
A model for flavonoid-mediated regulation of bamboo internode color variation.

Overall, our study outcomes displayed higher expression of *PvGL*, *PvUF7GT*, and *PvC12RT1* in culm, and their high expression also probably promoted prunin and rhoifolin accumulation. Furthermore, we proposed a possible model of internode color regulation in bamboo (Figure 4). The higher expression of *PvGL*, *PvUF7GT*, and *PvC12RT1* genes possibly enhances the accumulation of the prunin and rhoifolin in culm, and both metabolites may develop yellow culm in bamboo. This study may pave the foundation for the genetic breeding of ornamental bamboo in the future.

## Data Availability Statement

The original contributions presented in the study are publicly available. This data can be found here: National Center for Biotechnology Information (NCBI) BioProject database under accession number GSE157799.

## Author Contributions

Q-XQ conceived the project and designed the experiment. DH and MFA designed the experiments and reviewed the manuscript. H-TW collected samples, extracted RNA, processed the data, and wrote the original draft. H-WL, JZ, and J-LP performed the experiments. All authors contributed to the article and approved the submitted version.

## Conflict of Interest

The authors declare that the research was conducted in the absence of any commercial or financial relationships that could be construed as a potential conflict of interest.

## Publisher’s Note

All claims expressed in this article are solely those of the authors and do not necessarily represent those of their affiliated organizations, or those of the publisher, the editors and the reviewers. Any product that may be evaluated in this article, or claim that may be made by its manufacturer, is not guaranteed or endorsed by the publisher.

## Footnotes

1
https://www.mzcloud.org/

2
http://www.chemspider.com/

3
https://www.kegg.jp/kegg/

4
http://www.hmdb.ca/

5
http://www.lipidmaps.org/

6
https://www.ebi.ac.uk/metabolights/MTBLS3970

7
https://www.ncbi.nlm.nih.gov/geo/query/acc.cgi?acc=GSE157799

## Supplementary Material

Supplementary Figure 1 The overall workflow of this study.

Supplementary Figure 2 Pearson correlation coefficients between positive **(A)** and negative **(B)** quality control (QC) samples of the metabolome.

Supplementary Figure 3 Principal component analysis (PCA) analysis between positive **(A)** and negative **(B)** ionization mode of the metabolome.

Supplementary Figure 4 Summary of sample clustering. The completeness of transcripts was assessed using Benchmarking Universal Single-Copy Orthologs (BUSCO) **(A)**. PCA was performed on both sets (from the sulcus and culm) of samples based on gene expression profiles **(B)**.

Supplementary Figure 5 The volcano plot about gene expression levels about culm vs. sulcus.

Supplementary Figure 6 Functional annotation of the metabolites using the Kyoto Encyclopedia of Genes and Genomes (KEGG) database. Various metabolites were identified between positive **(A)** and negative **(B)** ionization modes in sulcus and culm of *P. violascens*.

Supplementary Figure 7 Functional classification of the transcriptome *via* Gene Ontology (GO) and Eukaryotic Orthologous Group (KOG) analysis. GO **(A)** and KOG **(B)** functional annotations in sulcus and culm in *P. violascens* transcriptome.

Supplementary Figure 8 Determination of differentially expressed genes (DEGs) and metabolites in sulcus and culm. Bubble diagrams of the KEGG enrichment of DEGs between sulcus and culm at the transcript level **(A)** and differential metabolites between both tissues at the metabolome level **(B)**.

Supplementary Figure 9 qRT-PCR analysis of *PvGL* **(A)**, *PvUF7GT* **(B)**, and *PvC12RT1* **(C)** expression between the sulcus and culm. Numbers represent three different repeats of bamboos. Significant differences were determined by one-way ANOVA with Duncan *post-hoc* test (*p* < 0.05).
