# Supplementary figures and images for: Metabolic Profiling and Transcriptome Analysis Reveal the Key Role of Flavonoids in Internode Coloration of Phyllostachys violascens cv. Viridisulcata
Source: Front Plant Sci. 2022 Jan 28;12:788895. doi: 10.3389/fpls.2021.788895 (PMC8832037; doi:10.3389/fpls.2021.788895)

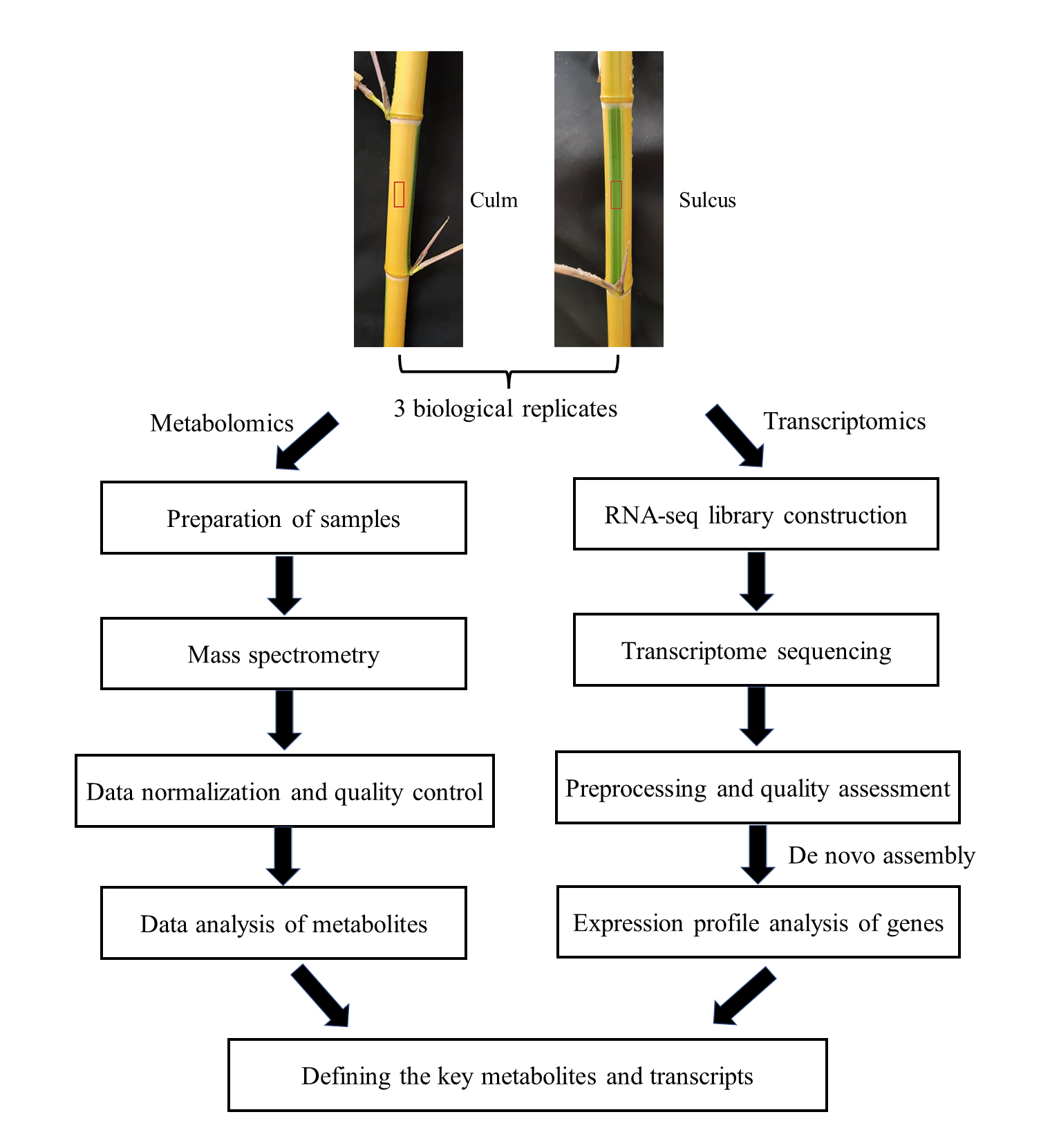

Supplement: Supplementary Figure 1 — The overall workflow of this study. [file Image_1.TIF]

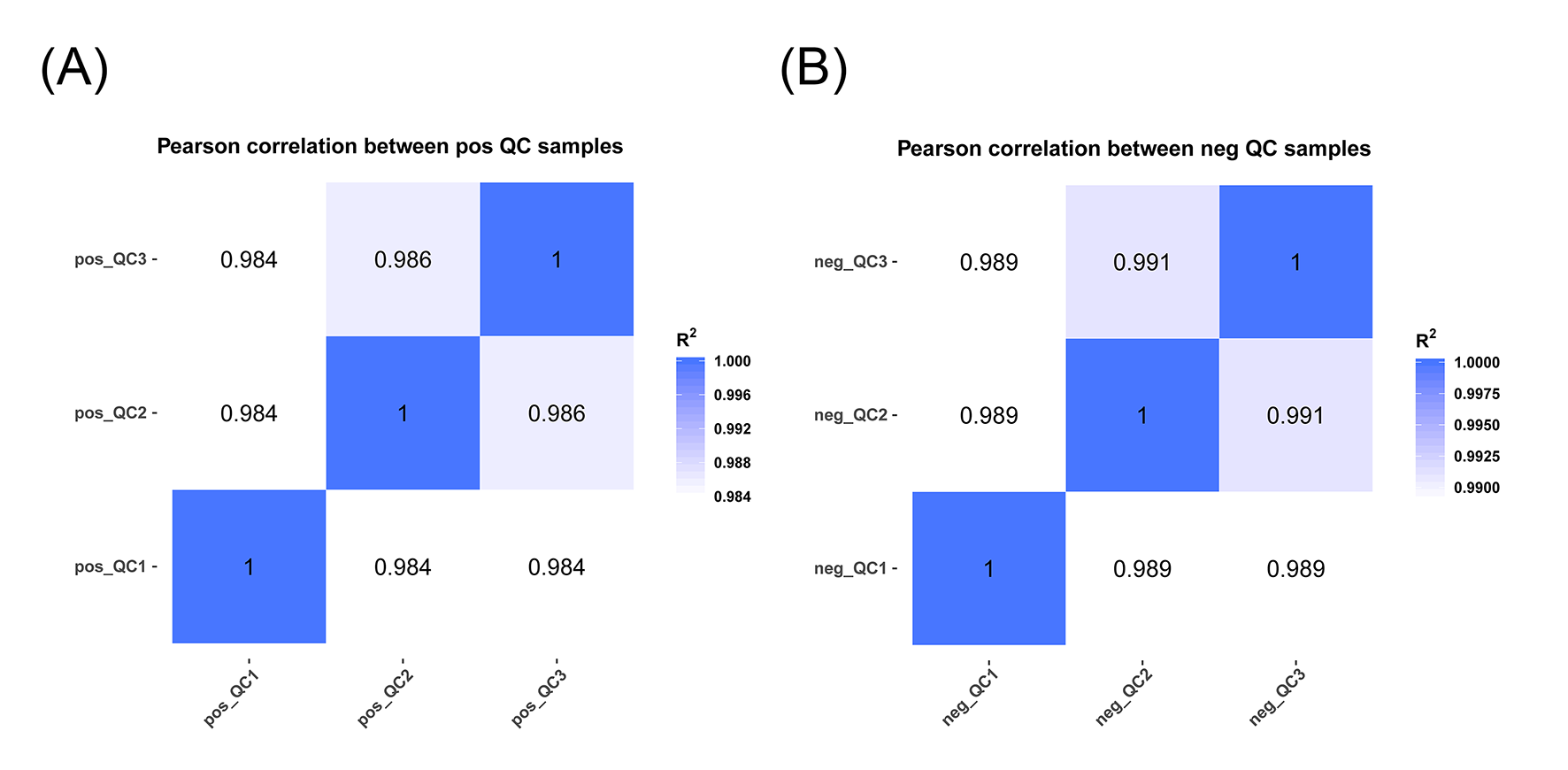

Supplement: Supplementary Figure 2 — Pearson correlation coefficients between positive (A) and negative (B) quality control (QC) samples of the metabolome. [file Image_2.TIF]

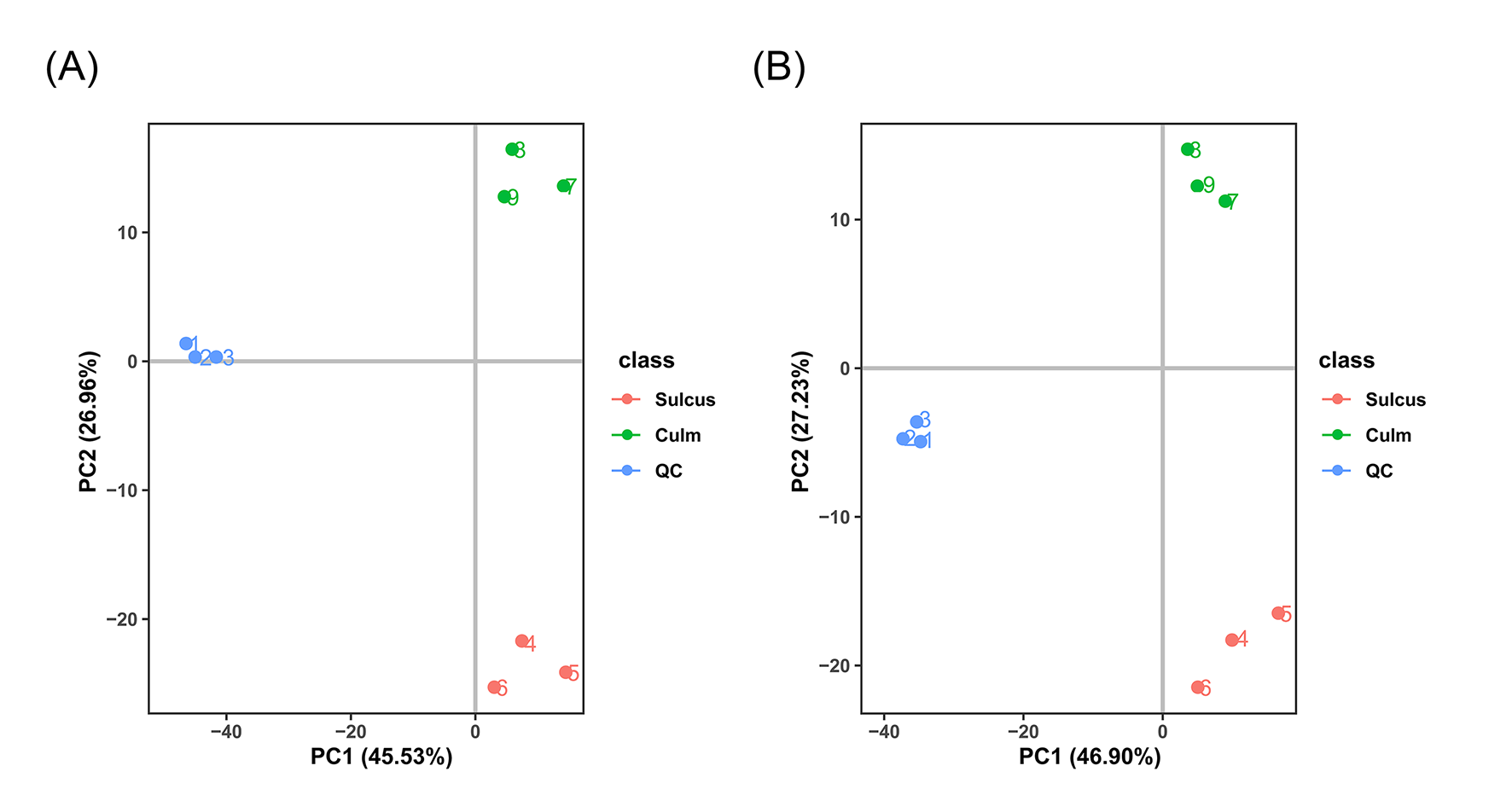

Supplement: Supplementary Figure 3 — Principal component analysis (PCA) analysis between positive (A) and negative (B) ionization mode of the metabolome. [file Image_3.TIF]

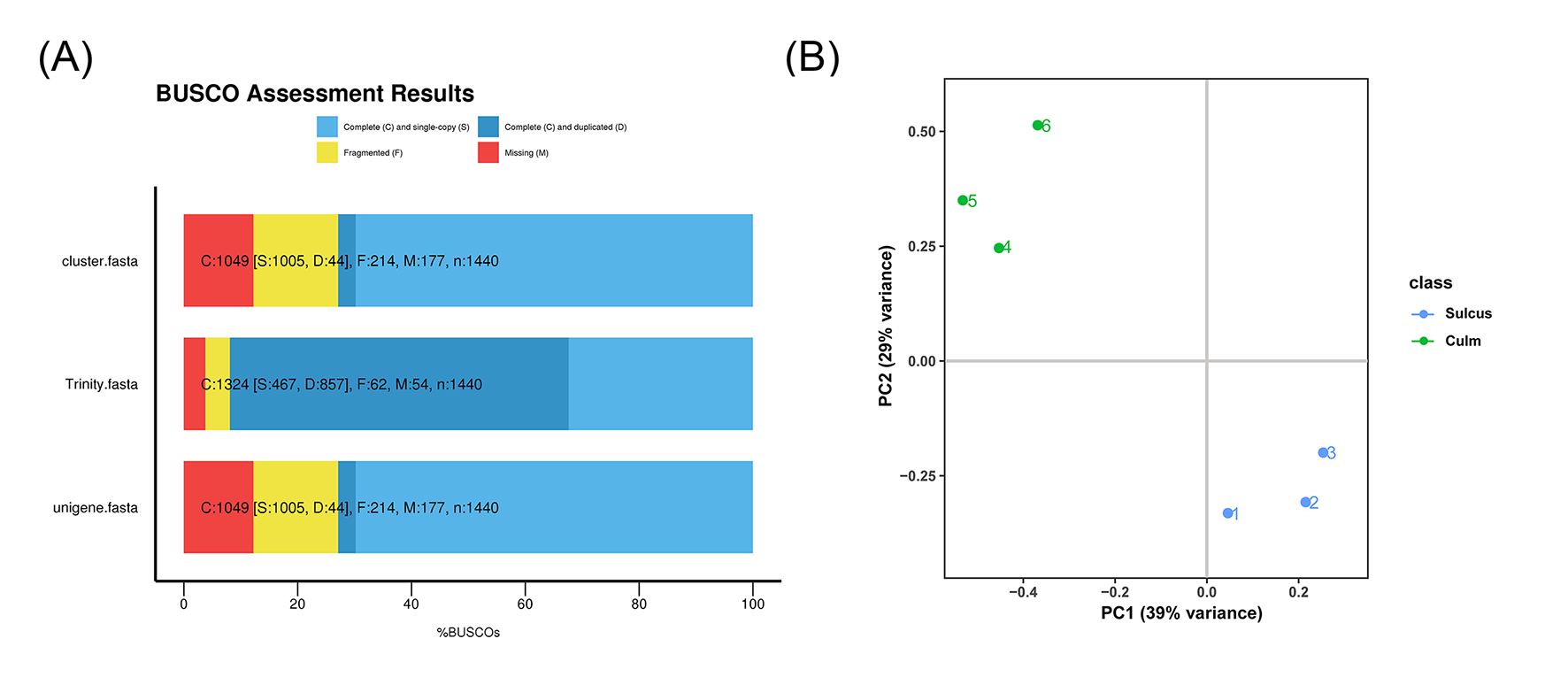

Supplement: Supplementary Figure 4 — Summary of sample clustering. The completeness of transcripts was assessed using Benchmarking Universal Single-Copy Orthologs (BUSCO) (A). PCA was performed on both sets (from the sulcus and culm) of samples based on gene expression profiles (B). [file Image_4.TIF]

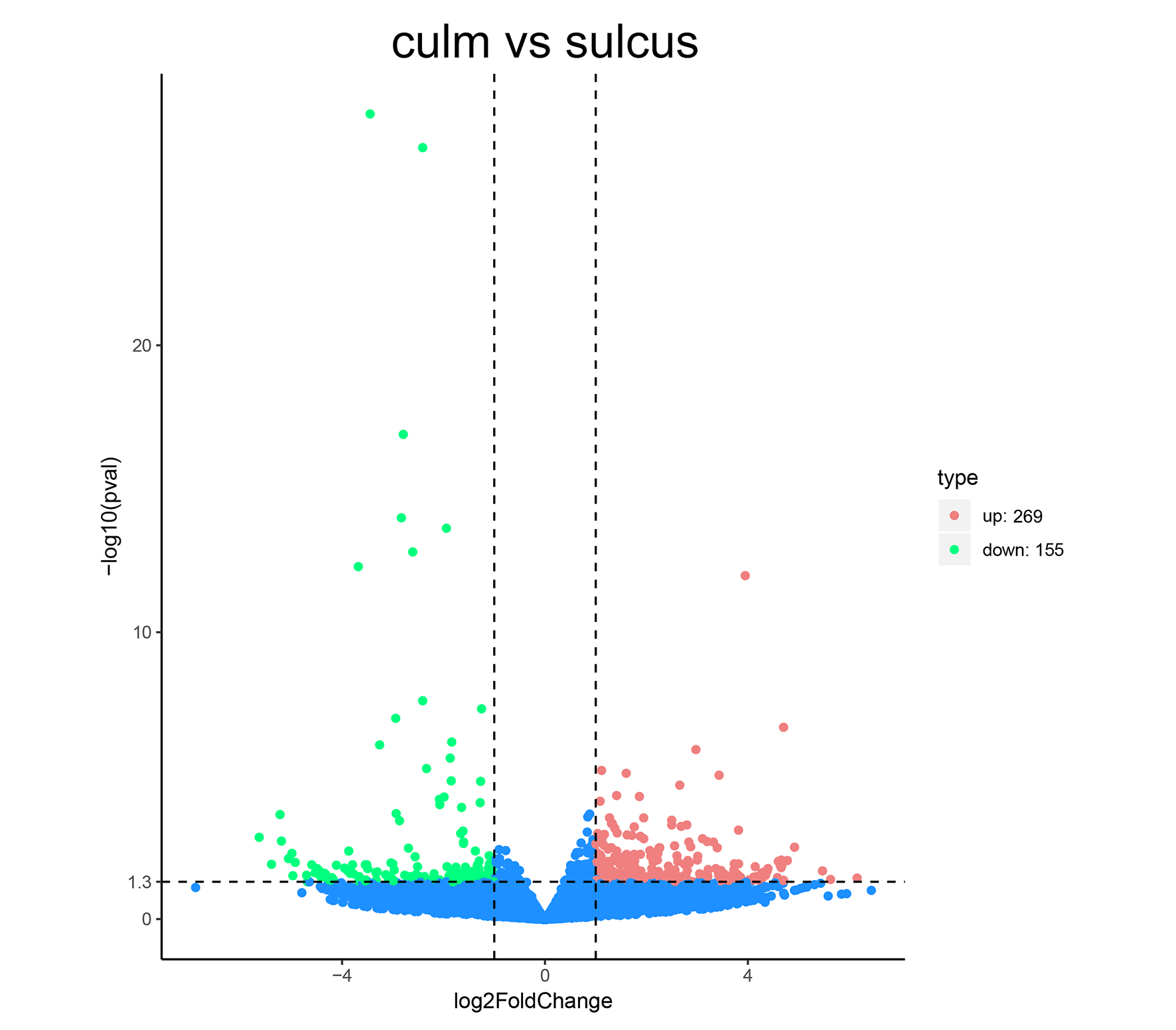

Supplement: Supplementary Figure 5 — The volcano plot about gene expression levels about culm vs. sulcus. [file Image_5.TIF]

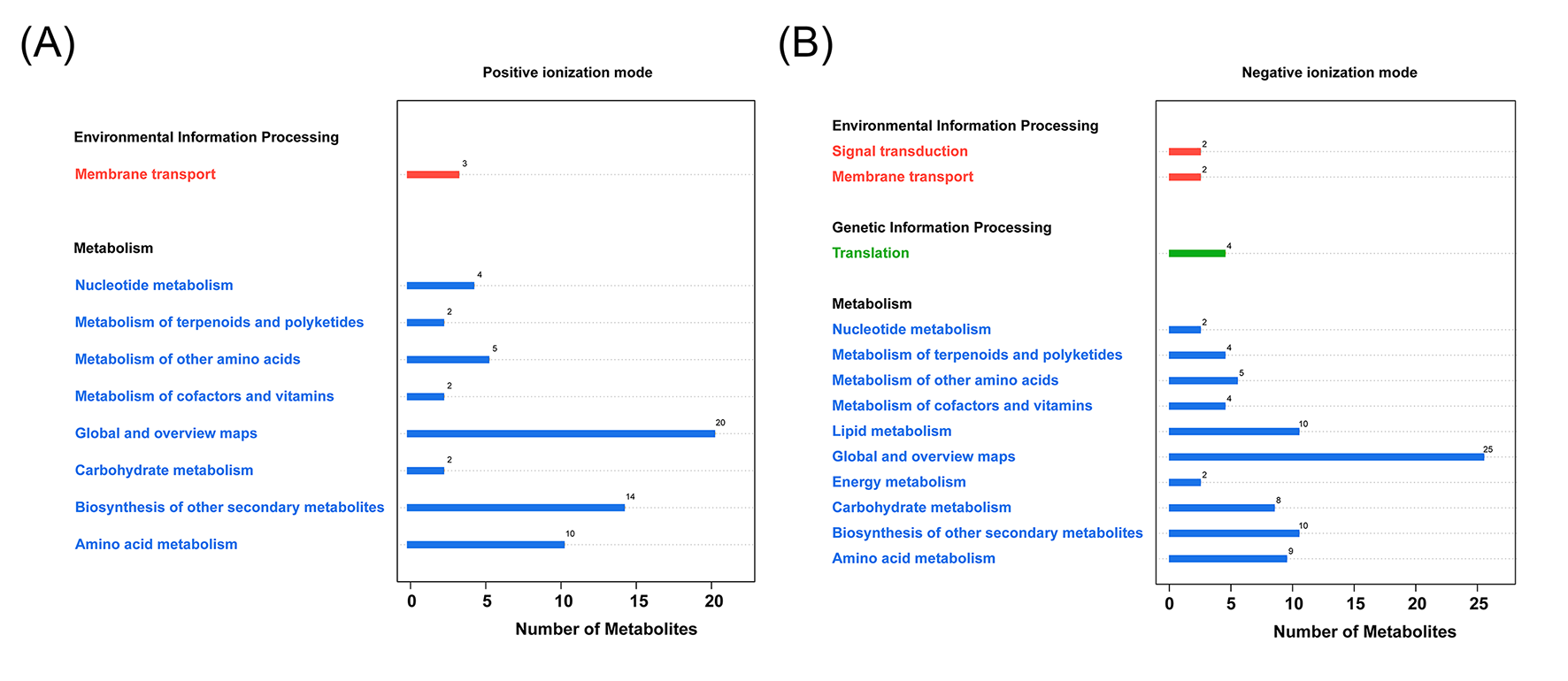

Supplement: Supplementary Figure 6 — Functional annotation of the metabolites using the Kyoto Encyclopedia of Genes and Genomes (KEGG) database. Various metabolites were identified between positive (A) and negative (B) ionization modes in sulcus and culm of P. violascens. [file Image_6.TIF]

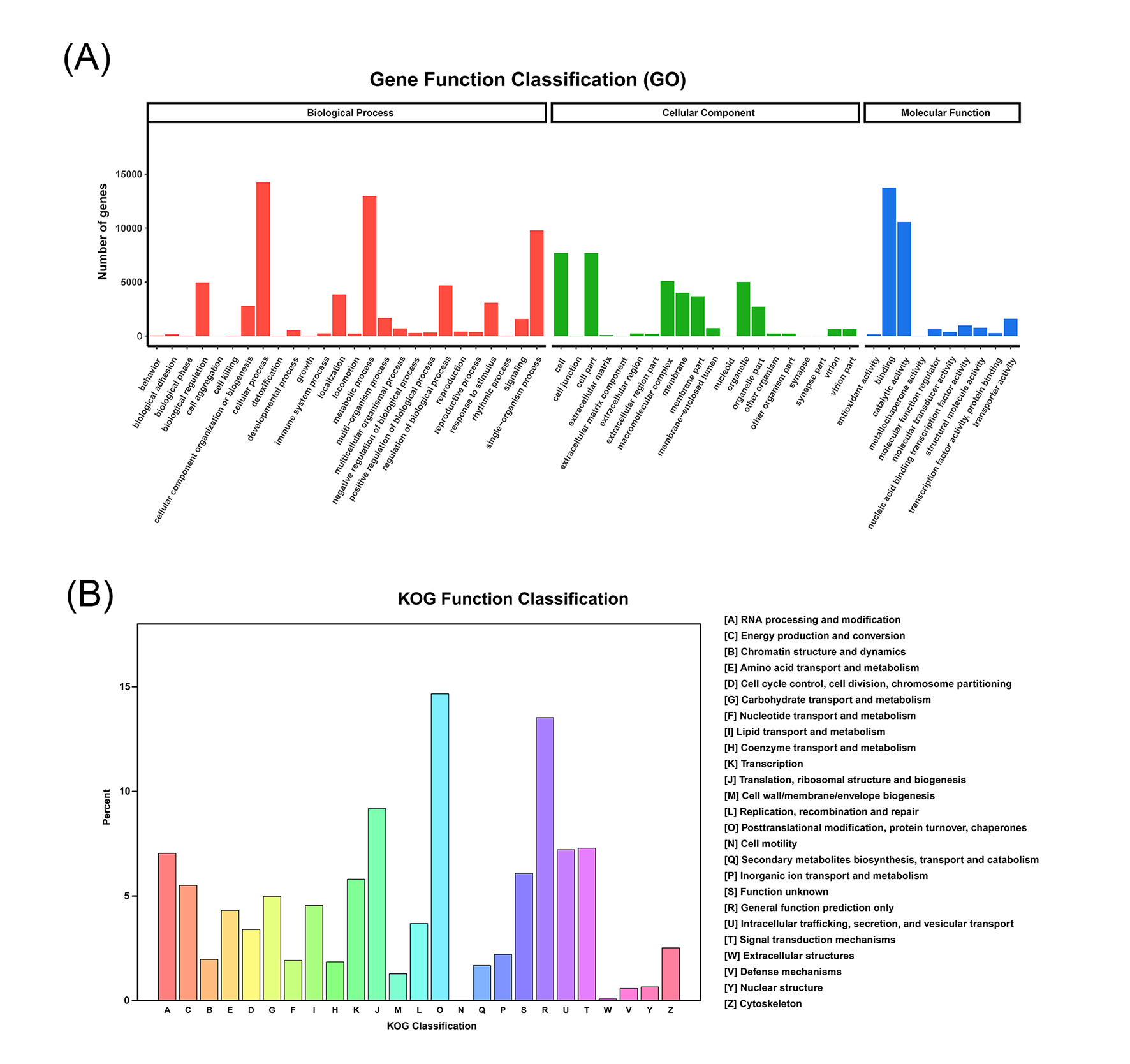

Supplement: Supplementary Figure 7 — Functional classification of the transcriptome via Gene Ontology (GO) and Eukaryotic Orthologous Group (KOG) analysis. GO (A) and KOG (B) functional annotations in sulcus and culm in P. violascens transcriptome. [file Image_7.TIF]

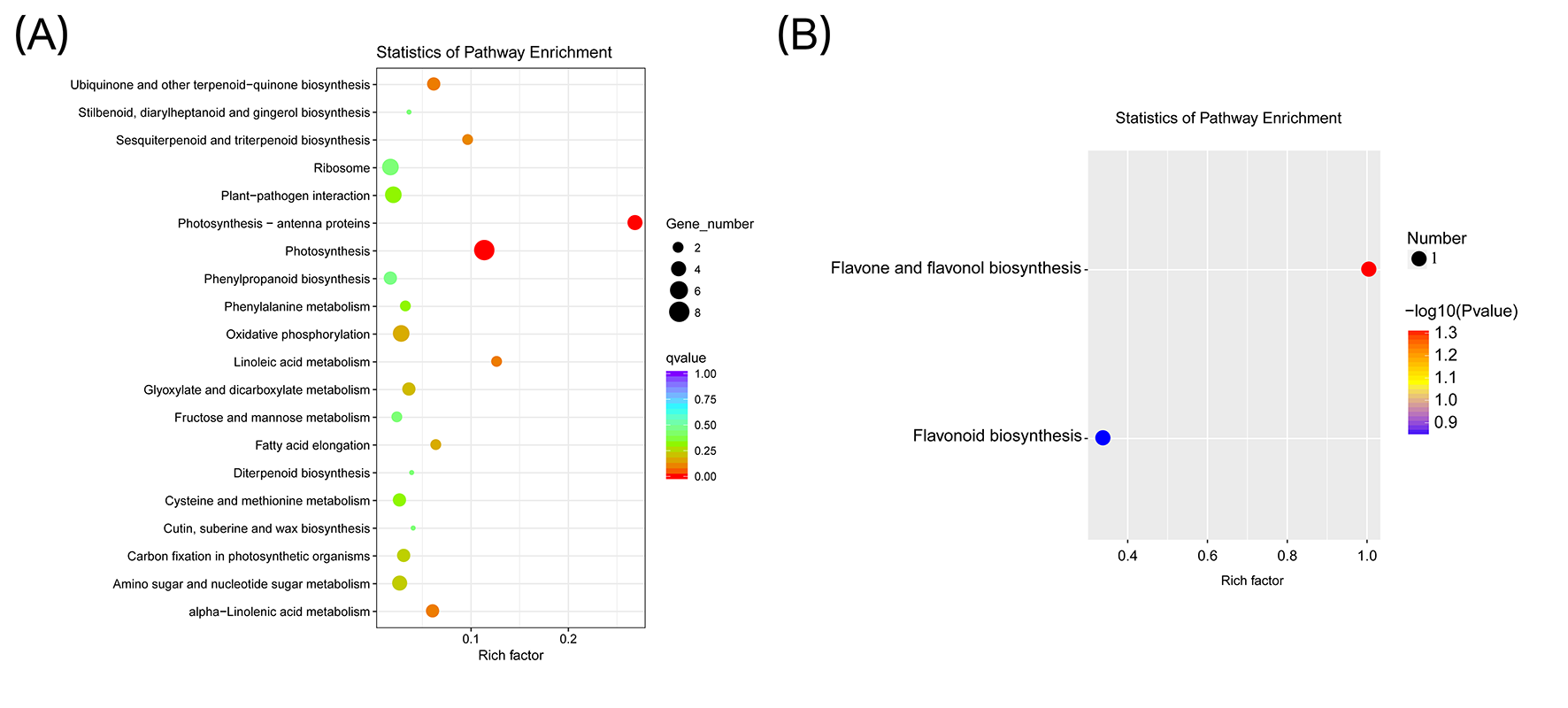

Supplement: Supplementary Figure 8 — Determination of differentially expressed genes (DEGs) and metabolites in sulcus and culm. Bubble diagrams of the KEGG enrichment of DEGs between sulcus and culm at the transcript level (A) and differential metabolites between both tissues at the metabolome level (B). [file Image_8.TIF]

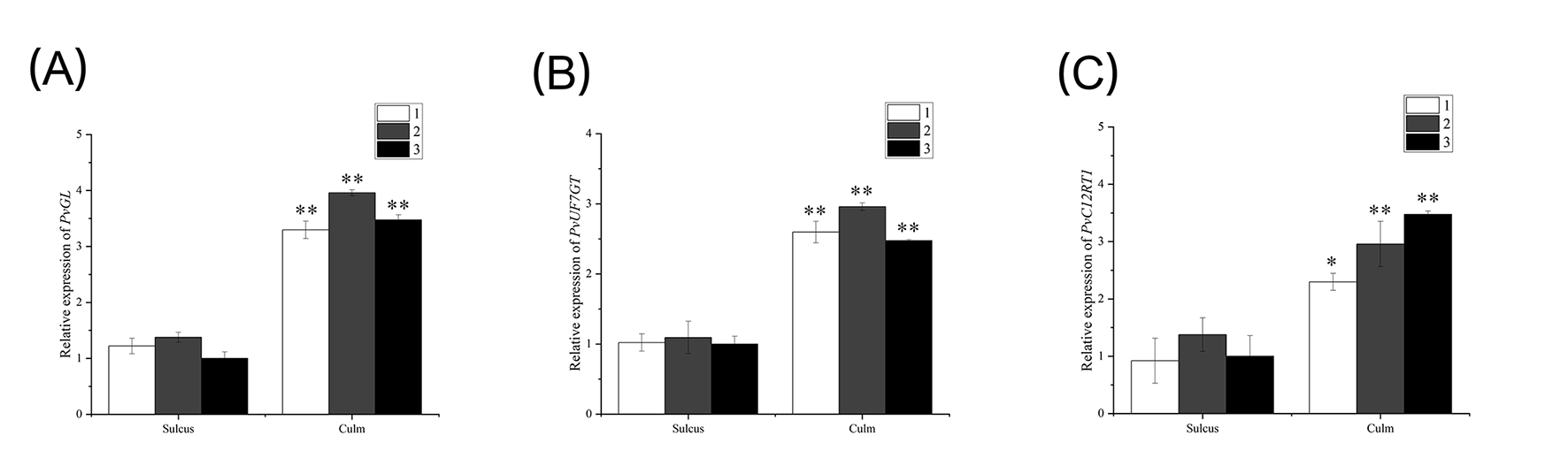

Supplement: Supplementary Figure 9 — qRT-PCR analysis of PvGL (A), PvUF7GT (B), and PvC12RT1 (C) expression between the sulcus and culm. Numbers represent three different repeats of bamboos. Significant differences were determined by one-way ANOVA with Duncan post-hoc test (p < 0.05). [file Image_9.TIF]
